# Load- and velocity-specific adaptations to lower-body maximal strength training (MST), hypertrophy training (HT) and explosive strength training (EST)

**DOI:** 10.1007/s00421-026-06156-2

**Published:** 2026-02-12

**Authors:** Glenn Trane, Olav Melhus Gomo, Stine Pedersen Bøtun, Jan Helgerud, Runar Jakobsen Unhjem

**Affiliations:** 1 Faculty of Education and Arts, Nord University, Universitetsalléen 11, 8026 Bodø, Norway; 2 Faculty of Medicine and Health Sciences, Norwegian University of Science and Technology, Trondheim, Norway; 3 Medical Rehabilitation Clinic, Treningsklinikken, Trondheim, Norway

**Keywords:** Counter movement jump (CMJ), Dual x-ray absorptiometry (DXA), Explosive strength training (EST), Hypertrophy training (HT), Maximal strength training (MST), Rate of force development (RFD), Squat, Squat jump (SJ)

## Abstract

**Purpose:**

We compared the effects of three strength training methods on squat performance; maximal strength training (MST), hypertrophy training (HT) and explosive strength training (EST). MST and EST were workload-matched, whereas HT was performed at higher volume.

**Methods:**

Fifty-eight moderately trained males (*n* = 29) and females (*n* = 29) were allocated to (1) MST-4 × 4 repetitions squats at ≥ 85% of one repetition maximum (1RM), (2) HT-3 × 8-12 repetitions squats at ∼70–80% of 1RM, (3) EST-4 × 6-7 unloaded squat jumps (SJ), (4) Control (CON). MST, HT and EST performed three sessions/week for eight weeks. Squat 1RM strength, SJ rate of force development (RFD) at 50%, 30% and 0% of squat 1RM (RFD_SJ50%_, RFD_SJ30%_ and RFD_SJ0%_), SJ- and counter movement jump (CMJ)-height and leg lean mass were examined.

**Results:**

MST and HT improved squat 1RM (+ 20.7% and + 18.2%) more than EST (+ 10.9%) (all *p* ≤ 0.01) and CON (all *p* ≤ 0.001). MST and HT improved RFD_SJ50%_ (+ 21.7% and + 14.3%, all *p* ≤ 0.001), RFD_SJ30%_ (+ 17.8% and 14.0%, *p* ≤ 0.001–0.01) and RFD_SJ0%_ (+ 12.3% and + 11.4%, *p* ≤ 0.01–0.05), while EST and CON did not. EST improved SJ-height (+ 2.2 cm) and CMJ-height (+ 1.7 cm) (all *p* ≤ 0.001) to a similar extent as MST (+ 2.2 cm and + 2.3 cm, all *p* ≤ 0.001) and HT (+ 2.0 cm and + 1.6 cm, all *p* ≤ 0.01).

**Conclusion:**

MST and HT led to improvements across all measured squat and jumping variables, whereas EST improved jump performance under conditions of low external load and high velocity. Improved jumping performance following MST and HT seems to rely largely on changes in muscle strength, whereas for EST, other mechanisms not assessed in this study may play a greater role.

**Supplementary Information:**

The online version contains supplementary material available at 10.1007/s00421-026-06156-2.

## Introduction

External load (kg) and concentric velocity (m ∙ s^− 1^) are interdepended strength training variables which are commonly manipulated to target specific neuromuscular and performance adaptations (Kraemer and Ratamess 2004; Ratamess et al. 2009; Schoenfeld et al. 2021a). If strength training is performed to increase maximal strength and velocity against high resistance, broad consensus exists that heavy loads should be used (Campos et al. 2002; Cormie et al. 2007a, 2010a, 2011; Haff and Nimphius 2012; Harris et al. 2000, 2008; Heggelund et al. 2013; Jones et al. 2001; Kraemer and Ratamess 2004; McBride et al. 2002; Schoenfeld et al. 2016, 2021a, b; Suchomel et al. 2016, 2018; Wilson et al. 1993). If the purpose is to increase velocity against a relatively low load, which is the case for many athletic capabilities, the optimal training method is not immediately evident (Behm et al. 2025; Cormie et al. 2011). The concept of velocity-specificity implies that strength training should be performed at concentric velocities that mimics the velocity of the task you want to improve, to maximize performance in explosive tasks (Behm and Sale 1993a, b; Behm et al. 2025). By deduction from Newton’s second law of motion, others advocate that the strength training regime that produces the largest gain in maximal strength would also produce the largest improvement in velocity against low loads, since acceleration is directly proportional to the force applied against a constant mass (Hoff and Helgerud 2004; Trane et al. 2025).

Recently, we investigated load- and velocity-specific adaptations to three training regimes in the upper-body: (1) explosive strength training (EST); 4 sets x 6–7 repetitions bench press throws at 40% of one repetition maximum (1RM), with sets terminated far from muscular failure, (2) hypertrophy training (HT); 3 sets x 8–12 repetitions bench press at 70–80% of 1RM, with most sets performed with 1–2 repetitions in reserve (RIR), approaching muscular failure, and (3) maximal strength training (MST); 4 sets x 4 repetitions bench press at ≥ 85% of 1RM, also with most sets performed with 1–2 RIR (Trane et al. 2025). As expected, MST and HT were more effective than EST to improve maximal strength and velocities against high external resistance (60–80% of 1RM). Surprisingly, adaptive changes in maximal strength outweighed the importance of velocity-specificity to improve velocity against low and moderate loads. Specifically, MST increased concentric mean propulsive velocity against 40% of bench press 1RM more than EST, despite that this was the exact load at which EST trained. Similarly, rate of force development (RFD) at 50% of bench press 1RM increased more after MST (+ 58%) and HT (+ 39%) compared to CON, whereas EST (+ 27%) did not improve RFD more than CON, again despite that 50% of 1RM was much closer to the training load of EST than MST and HT. Thus, for the bench press exercise, EST was not more effective than strength training with high loads to improve explosive strength at low to moderate loads, despite near perfect velocity-specificity. If anything, it was less effective. It is not clear whether these findings on upper-body performance are representative for the lower body.

Previous studies on lower-body strength training does not necessarily align with the ‘upper-body findings’ of Trane et al. (2025). In the literature EST are repeatedly shown to improve low-load athletic explosive performance (e.g. jumping height) (Cormie et al. 2007a, 2010a; Harris et al. 2000; Jones et al. 2001; McBride et al. 2002; Newton et al. 1999; Vissing et al. 2008; Wilson et al. 1993), and many argue that performance in explosive lower-body muscle actions can be effectively enhanced using both EST or strength training with high loads (Cormie et al. 2011; Haff and Nimphius 2012; Kawamori and Haff 2004; Kraemer and Newton 2000; Kraemer and Ratamess 2004; Suchomel et al. 2016, 2018). Lower-body RFD are consistently shown to increase following MST, HT and EST (Aagaard et al. 2002; Cormie et al. 2010a; Haglo et al. 2022; Heggelund et al. 2013; Helgerud et al. 2020; Newton et al. 1999; Støren et al. 2008; Tøien et al. 2018; Unhjem et al. 2015), and numerous studies report improvements in jumping performance ranging from 2–6 cm in squat jump (SJ)- and counter movement jump (CMJ)-height following MST, HT and EST (Cormie et al. 2010a; Harris et al. 2000; Jones et al. 2001; Vissing et al. 2008; Wilson et al. 1993). To our best knowledge, no previous studies have compared load- and velocity-specific adaptations to lower-body MST, HT and EST within the same framework, while at the same time contrasting lower- and upper body adaptations within the same sample (Trane et al. 2025).

Thus, the aim of this study was to compare lower-body strength training adaptations to 8 weeks of either; (1) 4 sets x 4 repetitions squat MST, (2) 3 sets x 8–12 repetitions squat HT or (3) 4 sets x 6–7 repetitions SJ EST, on squat 1RM, squat jump RFD at 50%, 30% and 0% of 1RM (RFD_SJ50%_, RFD_SJ30%_ and RFD_SJ0%_), SJ- and CMJ-height and leg lean mass within the same sample as Trane et al. (2025). We hypothesized that MST would be more effective than HT and EST to improve squat 1RM, RFD_SJ50%_ and RFD_SJ30%_. Moreover, considering the ‘upper-body findings’ within the same sample (Trane et al. 2025), we hypothesized that MST and HT would increase explosive performance against lower resistance, i.e. SJ- and CMJ-height and RFD_SJ0%_, more than EST.

## Methods

### Participants

Seventy-four subjects volunteered to participate in the present investigation. Subjects were classified as non-strength trained, but recreationally active and moderately trained. Age (years), height (cm), body weight (kg) and squat 1RM to body weight ratio for all subjects pooled were 27 ± 4 years, 172 ± 10 cm, 73.6 ± 15.0 kg and 1.32 ± 0.30, respectively. The sex-specific descriptive characteristics for these variables by group are presented in Table 1. Participants were recruited to the project through advertisement on Nord university’s website and social media platforms, and presentations in university classes or other arenas of appropriate target demographics. Inclusion in the study required; at least once weekly participation in sports or other recreational activities, age of ≥ 18 or ≤ 35 years, and being in good health with no contra-indications for strength training (Kittilsen et al. 2021). Illness exceeding ≥ 7 days over the course of the study, or adherence in less than 20 out of 24 training sessions led to exclusion from the current investigation. Subjects were instructed to maintain their usual activity levels, and refrain from excessive amounts of individual strength training outside the study during the training period, to limit interference. Written informed consents were collected from all subjects prior to the study. The consent form provided subjects with in-depth information about testing- and training-procedures and stated that participants were allowed withdraw from the study at any time, without any specific reason.

**Table 1:** Sex-specific descriptive subject’s characteristics

	All (*n* = 58)		MST (*n* = 16)		HT (*n* = 15)		EST (*n* = 14)		CON(*n* = 13)	
	Pre-test		Pre-test		Pre-test		Pre-test		Pre-test	
	Males (*n* = 29)	Females (*n* = 29)	Males (*n* = 8)	Females (*n* = 8)	Males (*n* = 9)	Females (*n* = 6)	Males (*n* = 7)	Females (*n* = 7)	Males (*n* = 5)	Female(*n* = 8)
Distribution (%)	50	50	50	50	60	40	50	50	38	62
Age (Years)	27±5	27±4	29±5	26±4	28±6	30±4	25±5	26±3	26±4	27±3
Height (cm)	180±8	165±5	179±7	168±5	183±9	166±5	179±10	164±4	175±5	164±5
Body weight (kg)	81.5±14.6	65.6±10.6	85.4±14.0	68.2±16.4	80.8±12.8	65.6±8.3	80.8±15.4	65.5±9.6	78.1±19.2	63.1±6.0
Squat 1RM (kg) to body weight ratio	1.44±0.27	1.20±0.29	1.41±0.30	1.12±0.33	1.49±0.29	1.23±0.25	1.43±0.27	1.12±0.27	1.42±0.31	1.34±0.30

Data are presented as mean ± SD. 1RM: one repetition maximum; MST: maximal strength training; HT: hypertrophy training; EST: explosive strength training; CON: control

### Ethical considerations

The Norwegian Knowledge Sector’s Service Provider (SIKT) approved the data storage procedures of the present investigation (SIKT reg. 393314). The regional ethical committee of health research (REK) evaluated the experimental protocol and concluded we were not obligated to submit the project for ethical approval for patient research (REK reg. 606483). The study was carried out in accordance with general ethical guidelines of Nord University and the Helsinki Declaration (World Medical Association 2013).

### Timeline and study design

This study is the second in a series of three from an extensive exploratory interventional trial where we aim to examine upper- and lower-body strength- and performance-adaptations between three different strength training methods: MST, HT and EST. In the first study, we examined upper-body training adaptations (Trane et al. 2025), whereas the present study examines lower-body training adaptations. The third study (currently unpublished) emphasizes the impact of combining sprint training with various strength training methods on complex athletic performance, including changes in 0–25-meter sprint running performance, endurance-related metrics and anaerobic performance. In the week leading up to pre-testing, all subjects met for an information presentation to sign written informed consent and receive detailed information about the experimental procedure. The presentation was immediately followed by an introductory session to familiarize all subjects to testing- and training-exercises. Familiarization was caried out to limit the influence of the learning effect, and make sure all subjects were able to perform lower-body exercises without any major restrictions.

Subjects were tested for three days at both pre- and post-test, respectively. Test day 1 included assessment of leg lean mass and body weight with dual-energy x-ray absorptiometry (DXA). Test day 2 consisted of testing of squat 1RM, while test day 3 consisted of testing of SJ- and CMJ-height and RFD_SJ50%_, RFD_SJ30%_ and RFD_SJ0%_, respectively. All testing-days were separated with at least two days of rest. Subjects were told to meet in a well-rested and > 2 h fasting condition for the DXA-analysis on test day 1 and instructed to steer clear of any vigorous exercise within a 24-hour time-window, avoid eating large meals and only drink water to stay properly hydrated within ≤ 2 h prior to testing day 2 and 3 (Kittilsen et al. 2021).

Randomization was carried out using a randomization software tool after test day 3 at pre-test. We equally randomized subjects into one of four groups: MST, HT, EST or control (CON), through block randomization based on their sex (i.e., males and females) and squat 1RM (kg) to body weight (kg) ratio (Bland 2015, p.10). Squat 1RM was selected as the most widely used representation of subject’s lower-body strength level in research (Suchomel et al. 2018). Randomization was followed by an 8-week training intervention including three weekly sessions for all training groups (MST, HT and EST) or two weekly sessions for CON.

### Testing equipment and procedures

#### Test day 1

##### Dual x-ray absorptiometry (DXA) - Leg lean mass and body weight

The first testing day was conducted at an external third-party location (Aspmyra stadion, Bodø, Norway) for the assessment of leg lean mass and body weight using GE Lunar Prodigy DXA (GE HealthCare, Madison, Wisconsin, USA). The pertaining software system enCORE software version 16.0 (GE HealthCare, Madison, Wisconsin, USA) was used by the test-leader to perform standardized DXA-scans in accordance with the manufacturers guidelines. Participants were informed to wear light clothing and remove any metal accessories before testing. The same individual test-leader (SPB) was responsible for conducting all DXA-scans and subsequent analysis in accordance with standardized procedures (Bazzocchi et al. 2016). Subjects were instructed to lay supine while the DXA machine scanned the body with a low dose radiation. To determine changes in lower-body muscular hypertrophy, specific assessment of leg lean mass (g) was detected automatically but manually adjusted by the test-leader with the software system before extracting the data, whereas body weight was derived from whole-body assessments. Leg lean mass included the entire foot, calf, and thigh, with the cutoff defined by a diagonal line crossing the femoral head and striking the lateral side of the pelvis (os ilium and os ischii). The remaining DXA data from whole-body and upper-body assessments within the same sample were previously presented in another study (Trane et al. 2025). Whole-body DXA-scans, including segmental lower-body assessments of leg lean mass, demonstrate excellent test-retest reliability, with intraclass correlation coefficients (ICC) ≥ 0.98 and coefficients of variation (CV) between 1 and 3% (Hart et al. 2015; Hind et al. 2011).

#### Test day 2

##### Squat one repetition maximum (1RM)

The second testing day was conducted at our research laboratory at Nord university (Bodø, Norway). Subjects performed a general warmup including 10 min cycling at a self-determined low intensity, followed by a specific warmup procedure prior to testing squat 1RM. Squat 1RM was assessed using a Hammer Strength HD Elite Power Rack (Life fitness, Inc, Rosemont, Illinois, USA) and Eleiko IPF calibrated weight plates and barbell (Eleiko AB, Halmstad, Sweeden). No accessories (e.g. lifting belt or knee sleeves) were allowed during testing. All squat 1RM-tests were carried out by the same test-leader (OMG), currently engaged as High-Performance Director of the Norwegian Powerlifting Federation. The specific warmup involved three to five sets of ≤ 10 repetitions at increasing submaximal loads ranging from ∼30 to 80% of 1RM, determined from subjects estimated 1RM at baseline. All warmup sets were separated by 1–3-minute breaks between each set. The squat was initiated in an upright position, with a loaded barbell at the upper portion of the subjects back. Subjects were instructed to take a deep breath to create a high intra-abdominal pressure, before the weight was lowered in a controlled manner in the eccentric portion of the movement to a 90 degree knee angle, followed a short distinct stop of approximately one second in the bottom position, before accelerating the weight ‘as fast as possible’ back to the starting position in the concentric phase (Behm and Sale 1993a; Brown and Weir 2001). Knee angle was visually assessed with a hand-held goniometer. A maximal of five single-repetition attempts were used to reach 1RM, separated by 3–5 min breaks to ensure complete rest. An Apple Ipad (Apple Inc, Cupertino, California, USA) was connected to an Vitruve linear position transducer (Vitruve, Madrid, Spain) to aid the test-leader to measure average velocity (m ∙ s^− 1^) and displacement (cm) at successful squat attempts, to objectively assess whether squat 1RM was at or close to maximal in terms of low average velocity of < 0.2 m ∙ s^− 1^ (González-Badillo & Sánchez-Medina, 2010), and ensure range of motion was maintained at each attempt although load increased. The testing loads started at 90% of an estimated 1RM, followed by stepwise increases of 2.5–10 kg until 1RM was reached. Squat 1RM was registered in kg as the highest load lifted for one repetition. Data from our laboratory demonstrate excellent test-retest reliability for the assessment of squat 1RM (ICC = 0.991, 95% confidence interval (CI) [0.975, 0.997], CV = 2 ± 2%), in line with Grgic et al. (2020).

#### Test day 3

The third testing day was conducted at the same research laboratory at Nord university (Bodø, Norway). Subjects completed an identical general warmup as on the second testing day, followed by testing SJ- and CMJ-height and RFD_SJ50%_, RFD_SJ30%_ and RFD_SJ0%_. All tests were carried out using an AMTI Accupower force plate, sampling at 1000 Hz (Advanced Mechanical Technology, Inc., Watertown, USA). SJ- and CMJ-height (cm) and force-time data for RFD_SJ50%_, RFD_SJ30%_ and RFD_SJ0%_ (N ∙ s^− 1^) calculations were obtained from the AMTI Accupower software installed on a PC connected to the force plate (Advanced Mechanical Technology, Inc., Watertown, USA). A Musclelab linear encoder (Ergotest Innovation AS, Stathelle, Norway) was connected to a light stick or barbell on the subjects back to aid the test-leader with displacement (cm), velocity (m ∙ s^− 1^) and power (W) data for all jumps.

##### Counter movement jump (CMJ)-height

Subjects were instructed to initiate the CMJ in a standing upright position with a light stick (< 1 kg) at subjects’ upper back, to avoid utilizing any arm swing in the jump. 1–3 warmup trials were performed before proceeding to testing maximal CMJ-height (cm). Subjects were instructed to jump ‘as high as possible’ in the concentric phase, by utilizing the counter movement from a quick descend to a self-determined depth in the eccentric phase. Subjects performed at least three successful attempts, separated with ∼1–2-minute breaks in between. The highest jump of three attempts was registered as jump height in centimetres (cm) and used in the data analysis. Data from our laboratory demonstrate excellent test-retest reliability for the assessment of CMJ-height (ICC = 0.992, 95% CI [0.988, 0.995], CV = 2 ± 2%), consistent with previous literature (Eythorsdottir et al. 2024).

##### Squat jump (SJ)-height

Similar warmup instructions to those given for the CMJ were provided to the subjects for the SJ. Subjects were instructed to start in the same upright initial starting position, with a < 1 kg stick at their upper back, to avoid any arm swing in the movement. Specific instructions were given to squat down eccentrically in a controlled manner, until they reached 90 degrees in the knee joint. Participants were instructed to avoid any rebound in the transition from the eccentric to the concentric part of the SJ, by performing a short distinct stop in the bottom position, immediately followed by instructions to ‘jump as high as possible’. The best of three jumps was registered as jump height (cm). Force-time data from this attempt was also used to calculate RFD_SJ0%_ in Newtons per second (N ∙ s^− 1^). Each attempt was separated by ∼1–2-minute breaks. Data from our laboratory demonstrate excellent test-retest reliability for the assessment of SJ-height (ICC = 0.988, 95% CI [0.981, 0.993], CV = 3 ± 2%), consistent with previous literature (Eythorsdottir et al. 2024).

##### Rate of force development (RFD) in SJ at 50%, 30% and 0% of squat 1RM (RFD_SJ50%_, RFD_SJ30%_ and RFD_SJ0%_)

Unloaded SJs to calculate RFD_SJ0%_, was directly followed by externally loaded SJs with 30% and 50% 1RM, to calculate RFD_SJ30%_ and RFD_SJ50%_, respectively. Similar technique instructions to those given for the unloaded SJs were given for the loaded SJs, with emphasis on accelerating the weight ‘as fast as possible’, along with ‘jump as high as possible’ (Behm and Sale 1993a; Maffiuletti et al. 2016). At least three valid attempts, separated by ≥ 2-minute breaks, were performed at each load. Force-time data from the best of three attempts at each load was used for the calculations of RFD_SJ50%_, RFD_SJ30%_ and RFD_SJ0%_ (N ∙ s^− 1^). RFD was calculated as Δforce (N)/Δtime (s) from 10% to 90% of peak force (N) in accordance with the methods outlined by Hoff et al. (2007). Peak force (N) was selected as the highest force obtained in the concentric phase of each SJ. Attempts with excessive rebound in the eccentric to concentric transition of the SJ were removed from analysis. 0% of peak force (i.e., onset of force) was determined as the first visible deviation of the force-time curve from the initial baseline-force level (Maffiuletti et al. 2016). Data from our laboratory demonstrate excellent test-retest reliability for the assessments of RFD_SJ0%_ (ICC = 0.962, 95% CI [0.928, 0.980], CV = 9 ± 7%), RFD_SJ30%_ (ICC = 0.957, 95% CI [0.914, 0.979], CV = 6 ± 5%), and RFD_SJ50%_ (ICC = 0.951, 95% CI [0.896, 0.978], CV = 6 ± 4%), in line with previous research (Dal Pupo et al. 2025; Spiering et al. 2011).

### Training intervention

All sessions were supervised at our training facilities at Nord university (Bodø, Norway). Adherence to training sessions was tracked by the supervisors using attendance logs. MST, HT and EST attended three weekly sessions for 8 weeks (24 sessions), whereas CON did not perform any structured lower-body strength training during the 8-week period. CON met twice a week (16 sessions) to perform 6 × 25-meter sprints. All strength training groups also performed the same sprint sessions as a part of two of the respective three weekly strength sessions. These sprint efforts were conducted to investigate the effect of integrating sprint training with strength training on complex athletic performance (e.g., sprint performance), as part of a separate publication that is not yet published. Load (kg) and repetitions were monitored carefully by the researchers to ensure the target repetition-zones were reached. MST and HT performed squats, while EST performed SJs. All training groups also performed bench press (MST and HT), or bench press throws (EST) after squats and SJs for the purpose of investigating upper-body training responses in a recently published study (Trane et al. 2025). All sessions started with a general warmup including low intensity cycling or treadmill walking/running for 10 min at ∼70% maximal heart rate, succeeded by specific warmup protocols described further in the following sections.

#### Sprint training (6 × 25-meter)

All sprints were carried out at a 25-meter sprint running track with an artificial turf surface. Sprints were performed directly after the general warmup twice per week in all groups. The specific warmup part consisted of three submaximal 25-meter sprints at increasing self-determined efforts ranging from ∼50%-, ∼70% to ∼90% of maximal 25-meter running speed, separated with ≥ 1–2-minute breaks in between. The specific warmup was followed by three maximal effort sprints, separated with 3-minute breaks. All sprint-starts were self-initiated from an upright staggered stance position. Subjects were encouraged by the researchers to ‘accelerate as fast as possible’, and ‘run as fast as possible’ from 0 to 25 m.

#### Maximal strength training (MST)

MST was conducted in line with well established procedures used to enhance maximal strength and RFD (Fimland et al. 2010; Haglo et al. 2022; Helgerud et al. 2020; Hoff and Helgerud 2004; Kittilsen et al. 2021; Trane et al. 2025; Tøien et al. 2018). The specific warmup included three sets of ≤ 10 repetitions at increasing submaximal loads from ∼30 to 80% of 1RM, separated by short ∼1–2-minute breaks. MST involved 4 sets x 4 repetitions at ≥ 85% of 1RM, separated by 3-minute breaks. Squats were initiated with a slow controlled eccentric phase (∼3 s) to a 90-degree knee angle, followed a distinct isometric pause in the bottom position (∼0.5 s) prior to the concentric phase, where emphasis was put on accelerating the weight ‘as fast as possible’ back to the starting position (Behm and Sale 1993a; Behm et al. 2025). The high load (≥ 85%) made the actual average movement velocity of each repetition low (< 0.5 m ∙ s^− 1^), although, the intent to move the weight ‘as fast as possible’ in the concentric phase was high. The load was increased linearly by 2.5–5 kg on the subsequent session every time subjects did manage to lift 5 repetitions in the fourth set with proficient technique (Fimland et al. 2010; Haglo et al. 2022; Helgerud et al. 2020; Hoff and Helgerud 2004; Kittilsen et al. 2021; Trane et al. 2025; Tøien et al. 2018).

#### Hypertrophy training (HT)

To effectively facilitate muscular hypertrophy, HT was carried out in line with the methods of Ratamess et al. (2009); Schoenfeld et al. (2021b). The specific warmup included three sets of ≤ 10 repetitions at increasing submaximal loads ranging from ∼30 to 70% of 1RM, separated by short ∼1–2-minute breaks. HT consisted of 3 sets x 8–12 repetitions at ∼70–80% of squat 1RM, separated by 3-minute breaks. Similar technique instructions as MST were used in the eccentric phase and bottom phase of the squat, whereas velocity in the concentric phase was self-determined, resulting in a low- to moderate actual average concentric velocity (< 0.75 m ∙ s^− 1^). However, fatigue made the intent to move the weight ‘as fast as possible’ a prerequisite in the final repetitions. Load was increased linearly by 2.5–5 kg on the subsequent session, every time subjects did manage to lift 12 repetitions in the third set with good technique (Heggelund et al. 2013). In accordance with the set end point suggestions of Schoenfeld (2020, p.131), HT was mostly carried out with ∼1–2 RIR to avoid large performance decrements on subsequent sets and allow for hypertrophic adaptation to occur, without accumulating too much fatigue, due to frequently approaching muscular failure in training (Carroll et al. 2019a, b; Lasevicius et al. 2022). However, subjects were allowed to draw some occasional sets to 0 RIR if this was necessary to reach the target repetition-zone in the final set.

#### Explosive strength training (EST)

EST involved ballistic-type body weight SJs, with instructions for the SJ technique like those provided on the third testing day. The specific warmup included 2–3 sets of 6–7 repetitions of SJs with a self-selected submaximal effort, separated by short breaks. EST consisted of 4 sets of 6 repetitions in week 1–4 and 4 sets of 7 repetitions in week 5–8, separated by 3-minute breaks, with emphasis on maximal intended velocity in every repetition (Behm and Sale 1993a; Behm et al. 2025). EST increased the number of repetitions from 6 to 7 from week 4 to 5 to compensate for the load increment in MST and HT. Load was kept constant throughout the intervention and chosen to maximize peak power output (PPO), referred to as the ‘optimal load’ in SJs (Bevan et al. 2010; Cormie et al. 2007b, 2008, 2011). This was supported by linear encoder data from pre-test, demonstrating higher PPO-values at SJ0% of 1RM (3799 ± 1206 W) compared to SJ30% of 1RM (3188 ± 901 W) and SJ50% of 1RM (2932 ± 784 W), respectively. Importantly, EST sets were terminated well before approaching muscular failure, as maintaining high average velocity (> 1.0 m ∙ s^− 1^) and PPO in the absence of excessive muscular fatigue was essential for EST.

#### Differences in training volume

Accounting for between-method differences in strength training volume is essential to compare training at different sections of the load-velocity profile and load-repetition continuum, with various RIR. Consistent with previous research, calculating concentric work in joules (J) for each method was considered the most appropriate option to compare training volume between squats in MST and HT, with SJs in EST (Vissing et al. 2008; Trane et al. 2025). Based on the Eq. (1), derived from Fleck and Kraemer (2014, p.7), we calculated concentric work (J) as:

1 $$\begin{array}{*{20}c} {Load{\text{ }}\left( N \right){\text{ }}\left( {\begin{array}{*{20}c} {External{\text{ }}weight} \\ {lifted{\text{ }}\left( {kg} \right)} \\ \end{array} {\text{ }} + {\text{ }}Body{\text{ }}weight{\text{ }}\left( {kg} \right)} \right)} \\ {Displacement{\text{ }}\left( m \right)} \\ \end{array} {\text{ }} = {\text{ }}Work{\text{ }}\left( J \right)$$

Thus, if an 80 kg subject squatted 100 kg for one repetition (∼1800 N) over a concentric distance of 55 cm; work (J) was calculated as 1800 N x 0.55 m = 990 J in one repetition, multiplied by sets x repetitions in the respective method (Fleck and Kraemer 2014, p.7; Trane et al. 2025; Vissing et al. 2008). Calculations from pilot testing revealed that HT involved a 1.5–2.5 times greater training volume compared to the workload-matched MST and EST. Moreover, all groups performed similar sprint-training volumes of 12 sprints per week and had similar 3-minute rest periods between all sets in strength training to offset the potential influence of these factors. Importantly, the sprint results are reported independently in another paper, which is currently unpublished.

### Statistical analysis

Statistics were calculated with the IBM SPSS statistics software, version 29 (Chicago, IL, USA). Figures were made using Graph pad prism, version 10.0 (La Jolla, CA, USA). One-way analysis of variance (ANOVA) and subsequent Fisher’s least significant difference (LSD) tests were used to detect differences between groups at baseline. Analysis of covariance (ANCOVA) and subsequent Fisher’s LSD tests were used to assess between-group differences in post-test values adjusted for pre-test values together with partial eta squared (η²) effect sizes reported for between-group comparisons. η² effect sizes were interpreted as small (*η²* = 0.01), moderate (*η²* = 0.06), and large (*η²* = 0.14) (Inglis et al. 2025; Lakens 2013). Within-group ∆ from pre- to post-test coupled with Cohen’s *d* effect sizes of significant within-group ∆ from pre- to post-test were assessed using paired samples t-tests. Cohen’s *d* effect sizes were presented along with 95% CI and interpreted as trivial (*d* = < 0.35), small (*d* = 0.35–0.80), moderate (*d* = 0.80–1.50) and large (*d* = > 1.50), according to (Rhea, 2004). Correlations were analysed with the Pearson correlation tests. The level of significance was defined as *p* ≤ 0.05. Data are presented as mean ± standard deviation (SD) unless otherwise stated.

ANCOVAs and Fisher’s LSD post hoc tests were selected as the most appropriate statistical approach in the three studies from this exploratory interventional trial (i.e., present study; Trane et al. 2025; third unpublished study) to address the leading research question of whether EST would lead to adaptations that could not be achieved to the same extent through MST, particularly in explosive performance outcomes such as RFD, velocity of movement and jump height. Because only small differences between MST and EST were expected based on previous literature (Behm et al. 2025; Cormie et al. 2011), this statistical approach provided the sensitivity required to detect such small between-group differences. Although the inclusion of HT and CON, to expand the context of our main research question, inevitably increased the number of between-group comparisons, no statistical adjustments for multiple comparisons were applied, as such corrections could have reduced the chance of detecting meaningful differences between MST (or HT) and EST. In particular, the higher statistical power and sensitivity of Fisher’s LSD post hoc tests were chosen over more conservative post hoc adjustments (e.g., Bonferroni correction), as the latter may increase the risk of Type II errors (i.e., false negatives) and potentially mask meaningful differences when expected between-group differences are small, although at the expense of a higher risk of Type I errors (i.e., false positives), especially when multiple comparisons are performed.

Accordingly, this should also be considered when interpreting the findings of the present study by separating confirmatory analyses, based on predefined primary outcome variables and hypotheses, from exploratory analyses. Specifically, the hypotheses and corresponding confirmatory outcome variables were: (1) that MST would be more effective than HT and EST in improving squat 1RM, RFD_SJ50%,_ and RFD_SJ30%_, and (2) that MST and HT would increase explosive performance against lower resistance, i.e. SJ- and CMJ-height and RFD_SJ0%_, more than EST. All other outcomes and corresponding between-group comparisons should be treated as exploratory and interpreted with caution.

## Results

58 subjects (29 males and 29 females) from the initial 74 who volunteered to participate in the study met all inclusion criteria and were included in the data analysis, which was conducted as a per-protocol analysis. Of the 74 subjects initially recruited, 16 either dropped out or were excluded from the study. Four dropped out due to unrelated injury, while two dropped out due to training-related injury. Specifically, one MST-subject experienced recurring back pain when squatting heavy and one CON-subject suffered strain injury from sprinting. One participant dropped out due to pregnancy, three dropped out due to time-constraints, while three did not state any reason. One subject was excluded from EST due to missing data, whereas two were excluded from CON, as they partook in 3–5 weekly strength training sessions apart from the study, in violation of the compliance guidelines. One female EST-subject was excluded from the single variables of RFD_SJ0%_ and ∆ in time at RFD_SJ0%_ due to inaccurate force curve measurements. The male- and female-distribution after dropouts and exclusion are presented in Table 1. No significant between-group differences at baseline were detected in any variables, except for RFD_SJ50%_ (N ∙ s^− 1^) and ∆ in time (s) at RFD_SJ50%_.

### Compliance

The compliance criterion of ≥ 20 sessions was achieved in all groups. Compliance to strength training was on average 22 ± 1 (95 ± 6%), 23 ± 1 (95 ± 5%) and 22 ± 1 (93 ± 6%) of 24 sessions in MST, HT and EST, respectively. Adherence to sprint training was on average 15 ± 1 (93 ± 8%), 15 ± 1 (92 ± 7%), 15 ± 1 (92 ± 8%) and 16 ± 1 (97 ± 5%) of 16 sessions in MST, HT, EST and CON, respectively.

### Squat one repetition maximum (1RM)

**Fig. 1:**
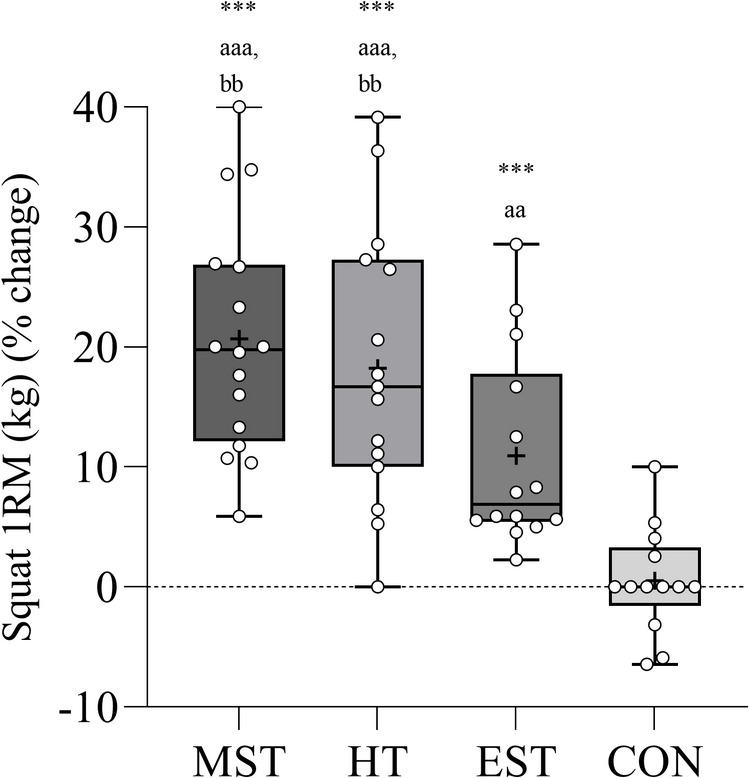
Percentage (%) change (∆) in squat one repetition maximum (1RM) (kg) from pre- to post-test in all training groups. The box plot displays individual values (○), mean (+), median (─), 1 st and 3rd quartiles, interquartile range, and minimum and maximum values. 1RM, One repetition maximum; MST, Maximal strength training; HT, Hypertrophy training; EST, Explosive strength training; CON, Control. Significant within-group ∆ from pre- to post-test (^***^p ≤ 0.001). Significant between-group difference in post-test value adjusted for pre-test value compared to CON (^aaa^ *p* ≤ 0.001, ^aa^ *p* ≤ 0.01) and EST (^bb^ *p* ≤ 0.01)

Squat 1RM (kg) improved more after MST, HT and EST compared to CON (all *p* ≤ 0.01 − 0.001), while MST and HT improved squat 1RM more than EST (all *p* ≤ 0.01). No difference in post-test squat 1RM (kg), adjusted for pre-test squat 1RM (kg) between MST and HT was found (*p* = 0.952). η² for the between-group comparisons was 0.479. Within groups, MST, HT, and EST increased squat 1RM (kg) by 17.8 ± 5.2 kg (*p* ≤ 0.001, *d* = 3.40, 95% CI [2.1, 4.7]), 17.7 ± 11.7 kg (*p* ≤ 0.001, *d* = 1.51, 95% CI [0.8, 2.3]) and 8.9 ± 5.5 kg (*p* ≤ 0.001, *d* = 1.62, 95% CI [0.8, 2.4]), respectively, from pre- to post-test, whereas no change in squat 1RM (kg) was observed in CON (Table 2; Fig. 1).

**Table 2:** Squat one repetition maximum (1RM) and DXA - Leg lean mass and body weight at pre- and post-test

	MST (*n* = 16) 8 males and 8 females		HT (*n* = 15) 9 males and 6 females		EST (*n* = 14) 7 males and 7 females		CON (*n* = 13) 5 males and 8 females	
	Pre-test	Post-test	Pre-test	Post-test	Pre-test	Post-test	Pre-test	Post-test
Squat 1RM (kg)	95.8 ± 31.5	113.6 ± 31.9^***^^aaa,bb^	103.8 ± 29.7	121.5 ± 31.9^***^^aaa,bb^	93.2 ± 27.5	102.1 ± 26.3^***^^aa^	94.4 ± 26.7	95.4 ± 29.2
Squat 1RM (kg) to body weight (kg) ratio	1.26 ± 0.33	1.49 ± 0.34^***^^aaa,bb^	1.39 ± 0.29	1.60 ± 0.29^***^^aaa,bb^	1.28 ± 0.31	1.40 ± 0.29^***^^aa^	1.37 ± 0.29	1.37 ± 0.31
DXA - Leg lean mass (g)	18,024 ± 3754	18,522 ± 3659^***^	18,435 ± 4009	19,079 ± 4050^***^^b^	17,415 ± 3696	17,613 ± 3853	16,459 ± 3416	16,794 ± 3430^**^
DXA - Body Weight (kg)	76.7 ± 17.6	77.5 ± 17.9	74.7 ± 13.3	75.8 ± 13.9^*^	73.2 ± 14.7	73.2 ± 14.9	68.9 ± 14.2	69.4 ± 13.9

Data are presented as mean ± SD. 1RM: One repetition maximum; DXA: Dual x-ray absorptiometry; MST: Maximal strength training; HT: Hypertrophy training; EST: Explosive strength training; CON: Control

Significant within-group ∆ from pre- to post-test (^***^p ≤ 0.001; ^**^p ≤ 0.01; ^*^p ≤ 0.05). Significant between-group difference in post-test value adjusted for pre-test value compared to CON (^aaa^ *p* ≤ 0.001, ^aa^ *p* ≤ 0.01) and EST (^bb^ *p* ≤ 0.01, ^b^ *p* ≤ 0.05)

Squat 1RM (kg) to body weight (kg) ratio improved more after MST, HT and EST compared to CON (all *p* ≤ 0.01 − 0.001), while MST and HT improved squat 1RM more than EST (all *p* ≤ 0.01). η² for the between-group comparisons was 0.487. Within groups, MST, HT, and EST increased squat 1RM (kg) to body weight (kg) ratio by 0.23 ± 0.09 (*p* ≤ 0.001, *d* = 2.56, 95% CI [1.5, 3.6]), 0.22 ± 0.13 (*p* ≤ 0.001, *d* = 1.68, 95% CI [0.9, 2.5]) and 0.12 ± 0.09 (*p* ≤ 0.001, *d* = 1.34, 95% CI [0.6, 2.1]), respectively, from pre- to post-test, whereas no change in squat 1RM (kg) was observed in CON (Table 2).

### DXA - Leg lean mass and body weight

**Fig. 2:**
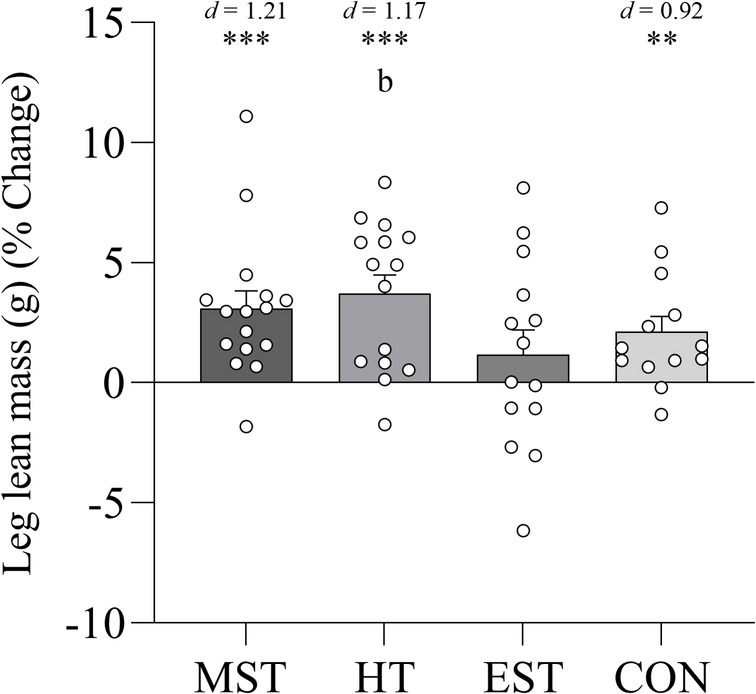
Percentage (%) change (∆) in leg lean mass (g) from pre- to post-test in all training groups. Data are presented as mean ± SE, along with Cohen’s *d* effect sizes. MST, Maximal strength training; HT, Hypertrophy training; EST, Explosive strength training; CON, Control. Significant within-group ∆ from pre- to post-test (^***^ *p* ≤ 0.001, ^**^ *p* ≤ 0.01). Significant between-group difference in post-test value adjusted for pre-test value compared to EST (^b^ *p* ≤ 0.05)

HT improved leg lean mass (g) more than EST (*p* ≤ 0.05), while no other between-group differences were found in post-test leg lean mass (g), adjusted for pre-test values. η² for the between-group comparisons was 0.090. Within groups, MST, HT and CON improved leg lean mass (g) by 498 ± 413 g (*p* ≤ 0.001, *d* = 1.21, 95% CI [0.6, 1.9]), 645 ± 553 g (*p* ≤ 0.001, *d* = 1.17, 95% CI [0.5, 1.2]) and 335 ± 364 g (*p* ≤ 0.01, *d* = 0.92, 95% CI [0.3, 1.6]), respectively, whereas EST did not change leg lean mass (g) (Table 2; Fig. 2). No between-group differences were found in post-test body weight (kg), adjusted for pre-test values. Body weight (kg) increased significantly from pre- to post-test within HT by 1.1 ± 1.8 kg (*p* ≤ 0.05, *d* = 0.64, 95% CI [0.1, 1.2]), whereas no within-group changes were apparent in MST, EST or CON, respectively (Table 2).

### Rate of force development (RFD) in squat jump (SJ) at 50%, 30% and 0% of 1RM (RFD_SJ50%_, RFD_SJ30%_, RFD_SJ0%_)

#### RFD_SJ50%_

**Fig. 3:**
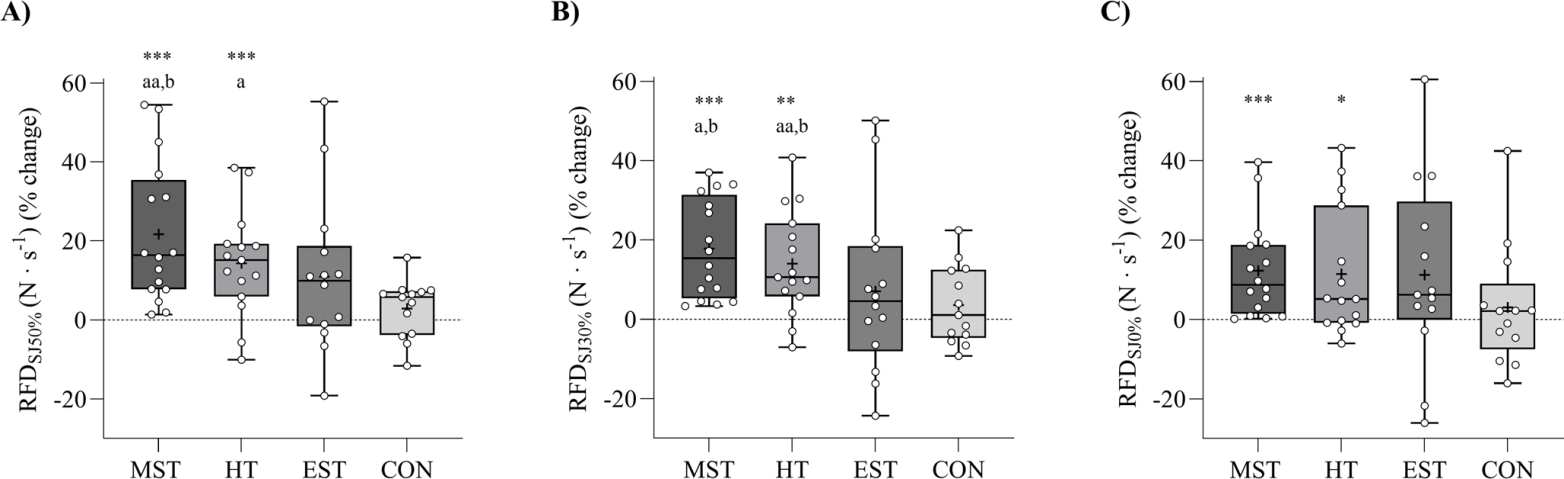
Percentage (%) change (∆) in **A** dynamic rate of force development (RFD) (N ∙ s^− 1^) in squat jump (SJ) at 50% of squat 1RM (RFD_SJ50%_), **B** dynamic rate of force development (RFD) (N ∙ s^− 1^) in squat jump (SJ) at 30% of squat 1RM (RFD_SJ30%_), and **C** dynamic rate of force development (RFD) (N ∙ s^− 1^) in squat jump (SJ) at 0% of squat 1RM (RFD_SJ0%_) from pre- to post-test in all training groups. Box plots display individual values (○), mean (+), median (─), 1 st and 3rd quartiles, interquartile range, and minimum and maximum values. RFD_SJ50%_, RFD_SJ30%_, RFD_SJ0%_, Dynamic rate of force development (RFD) in squat jump (SJ) at 50%, 30% and 0% of squat 1RM, respectively; MST, Maximal strength training; HT, Hypertrophy training; EST, Explosive strength training; CON, Control. Significant within-group ∆ from pre- to post-test (^***^p ≤ 0.001, ^**^p ≤ 0.01, ^*^ *p* ≤ 0.05). Significant between-group difference in post-test value adjusted for pre-test value compared to CON (^aa^ *p* ≤ 0.01, ^a^ *p* ≤ 0.05) and EST (^b^ *p* ≤ 0.05)

RFD_SJ50%_ (N ∙ s^− 1^) improved more after MST and HT compared to CON (*p* ≤ 0.01 and *p* ≤ 0.05, respectively), while MST improved more that EST (*p* ≤ 0.05). η² for the between-group comparisons was 0.168. Within groups, MST improved RFD_SJ50%_ (N ∙ s^− 1^) by 195 ± 154 N ∙ s^− 1^ (*p* ≤ 0.001, *d* = 1.27, 95% CI [0.6, 1.9]) and HT improved by 177 ± 168 N ∙ s^− 1^ (*p* ≤ 0.001, *d* = 1.06, 95% CI [0.4, 1.7]). Neither EST nor CON improved RFD_SJ50%_ from pre- to post-test (Table 3; Fig. 3A). No significant between-group differences in post-test peak force (N) from the RFD_SJ50%_-test (PF_SJ50%_), adjusted for pre-test values, were found. Within groups, PF_SJ50%_ (N) increased by 3.1 ± 2.2% after MST (*p* ≤ 0.001, *d* = 1.23, 95% CI [0.6, 1.9]), 2.8 ± 3.0% after HT (*p* ≤ 0.01, *d* = 1.04, 95% CI [0.4, 1.7]) and 2.5 ± 2.8% after CON (*p* ≤ 0.01, *d =* 0.88, 95% CI [0.2, 1.5]), respectively. No change in PF_SJ50%_ (N) was found after EST (*p* = 0.089, *d* = 0.41, 95% CI [−0.2, 0.9]) (Table 3).

**Table 3:** Dynamic rate of force development (RFD) in squat jump (SJ) at 50%, 30% and 0% of 1RM (RFD_SJ50%,_ RFD_SJ30%_, RFD_SJ0%_) at pre- and post-test

	MST (*n* = 16) 8 males and 8 females		HT (*n* = 15) 9 males and 6 females		EST (*n* = 14) 7 males and 7 females		CON (*n* = 13) 5 males and 8 females	
	Pre-test	Post-test	Pre-test	Post-test	Pre-test	Post-test	Pre-test	Post-test
RFD_SJ50%_								
RFD (N ∙ s^− 1^)	971 ± 297	1166 ± 333^***^^aa,b^	1209 ± 432^§a, b^	1386 ± 515^***^^a^	902 ± 268	987 ±290	944 ± 314	974 ±347
Peak force (N)	1869 ± 423	1926 ± 438^***^	1960 ± 442	2015 ± 456^**^	1779 ± 389	1819 ± 357	1749 ± 384	1791 ± 387^**^
∆ in time (s)	0.544 ± 0.081	0.452 ± 0.078^***^^aa^	0.481 ± 0.088^§c^	0.428 ± 0.099^**^^a^	0.533 ± 0.094	0.487 ± 0.088	0.522 ± 0.071	0.511 ± 0.082
External load (kg)	47.8 ± 15.8	47.8 ± 15.8	51.9 ± 14.8	51.9 ± 14.8	46.6 ± 13.7	46.6 ± 13.7	47.2 ± 13.3	47.2 ± 13.3
RFD _SJ30%_								
RFD (N ∙ s^− 1^)	1270 ± 465	1473 ± 485^***^^a, b^	1563 ± 553	1788 ± 686^**^^aa, b^	1255 ± 381	1307 ± 345	1311 ± 578	1330 ± 517
Peak force (N)	1702 ± 370	1757 ± 401^***^	1768 ± 363	1836 ± 399^***^^a, b^	1657 ± 374	1673 ± 352	1594 ± 355	1608 ± 367
∆ in time (s)	0.432 ± 0.081	0.366 ± 0.051^***^^a, b^	0.389 ± 0.072	0.349 ± 0.074^**^	0.417 ± 0.062	0.398 ± 0.075	0.407 ± 0.068	0.393 ± 0.059
External load (kg)	29.7 ± 9.5	29.7 ± 9.5	31.2 ± 8.9	31.2 ± 8.9	28.0 ± 8.2	28.0 ± 8.2	28.3 ± 8.0	28.3 ± 8.0
RFD _SJ0%_								
RFD (N ∙ s^− 1^)	2022 ± 821	2245 ± 865^***^	2698 ± 1643	3090 ± 2132^*^	2126 ± 673 ^(*N*=13)^	2319 ± 786 ^(*N*=13)^	2068 ± 961	2074 ± 856
Peak force (N)	1456 ± 314	1512 ± 349^**b^	1511 ± 348	1564 ± 352^**b^	1445 ± 341	1450 ± 295	1341 ± 314	1356 ± 296
∆ in time (s)	0.301 ± 0.077	0.270 ± 0.070^***^	0.272 ± 0.084	0.253 ± 0.087^*^	0.277 ± 0.050 ^(*N*=13)^	0.257 ±0.060 ^(*N*=13)^	0.274 ± 0.067	0.268 ± 0.059
External load (kg)	1.0 ± 0.0	1.0 ± 0.0	1.0 ± 0.0	1.0 ± 0.0	1.0 ± 0.0	1.0 ± 0.0	1.0 ± 0.0	1.0 ± 0.0

Data are presented as mean ± SD. RFD, Rate of force development, RFD_SJ50%_, RFD_SJ30%_, RFD_SJ0%_, Dynamic rate of force development (RFD) in squat jump (SJ) at 50%, 30% and 0% of 1RM squat, respectively; MST: Maximal strength training; HT: Hypertrophy training; EST: Explosive strength training; CON: Control

Significant within-group ∆ from pre- to post-test (^***^p ≤ 0.001, ^**^p ≤ 0.01, ^*^ *p* ≤ 0.05). Significant between-group difference in post-test value adjusted for pre-test value compared to CON (^aa^ *p* ≤ 0.01, ^a^ *p* ≤ 0.05) and EST (^b^ *p* ≤ 0.05). ^§^Significantly different from CON (^a^ *p* ≤ 0.05), EST (^b^ *p* ≤ 0.05) and MST (^c^ *p* ≤ 0.05) at baseline

#### RFD_SJ30%_

RFD_SJ30%_ (N ∙ s^− 1^) improved more in MST and HT compared to CON (*p* ≤ 0.05 and *p* ≤ 0.01, respectively) and EST (all *p* ≤ 0.05). η² for the between-group comparisons was 0.193. Within groups, MST improved RFD_SJ30%_ (N ∙ s^− 1^) by 203 ± 148 N ∙ s^− 1^ (*p* ≤ 0.001, *d* = 1.38, 95% CI [0.7, 2.1]), while HT improved by 224 ± 230 N ∙ s^− 1^ (*p* ≤ 0.01, *d* = 0.98, 95% CI [0.4, 1.6]). EST and CON did not improve RFD_SJ30%_ (N ∙ s^− 1^) significantly from pre- to post-test (Table 3; Fig. 3B). Only HT increased peak force (N) obtained in the RFD_SJ30%_-test (PF_SJ30%_) more compared to CON and EST, respectively (all *p* ≤ 0.05). η² for the between-group comparisons was 0.151. Within groups, MST and HT improved PF_SJ30%_ (N) by 3.1 ± 2.4% (*p* ≤ 0.001, *d* = 1.07, 95% CI [0.4, 1.7]) and 3.6 ± 3.5% (*p* ≤ 0.001, *d* = 1.03, 95% CI [0.4, 1.6]), respectively. No changes were found after EST or CON from pre- to post-test (Table 3).

#### RFD_SJ0%_

No between-group differences in post-test RFD_SJ0%_ (N ∙ s^− 1^), adjusted for pre-test values, were found. Within groups, MST and HT improved RFD_SJ0%_ (N ∙ s^− 1^) by 224 ± 208 N ∙ s^− 1^ (*p* ≤ 0.001, *d* = 1.08, 95% CI [0.4, 1.7]) and 392 ± 629 N ∙ s^− 1^ (*p* ≤ 0.05, *d* = 0.62, 95% CI [0.1, 1.2]), from pre- to post-test, while no changes were found in EST (*p* = 0.243) or CON (Table 3; Fig. 3C). Peak force (N) from the RFD_SJ0%_-test (PF_SJ0%_) improved more after MST and HT compared to EST (all *p* ≤ 0.05). η² for the between-group comparisons was 0.126. Within groups, PF_SJ0%_ (N) increased significantly in MST by 3.6 ± 4.1% (*p* ≤ 0.01, *d* = 0.82, 95% CI [0.2, 1.4]) and 3.6 ± 3.7% in HT (*p* ≤ 0.01, *d* = 0.90, 95% CI [0.3, 1.5]), while no changes were found in EST or CON (Table 3).

### Squat jump (SJ)- and counter movement jump (CMJ)-height

**Fig. 4:**
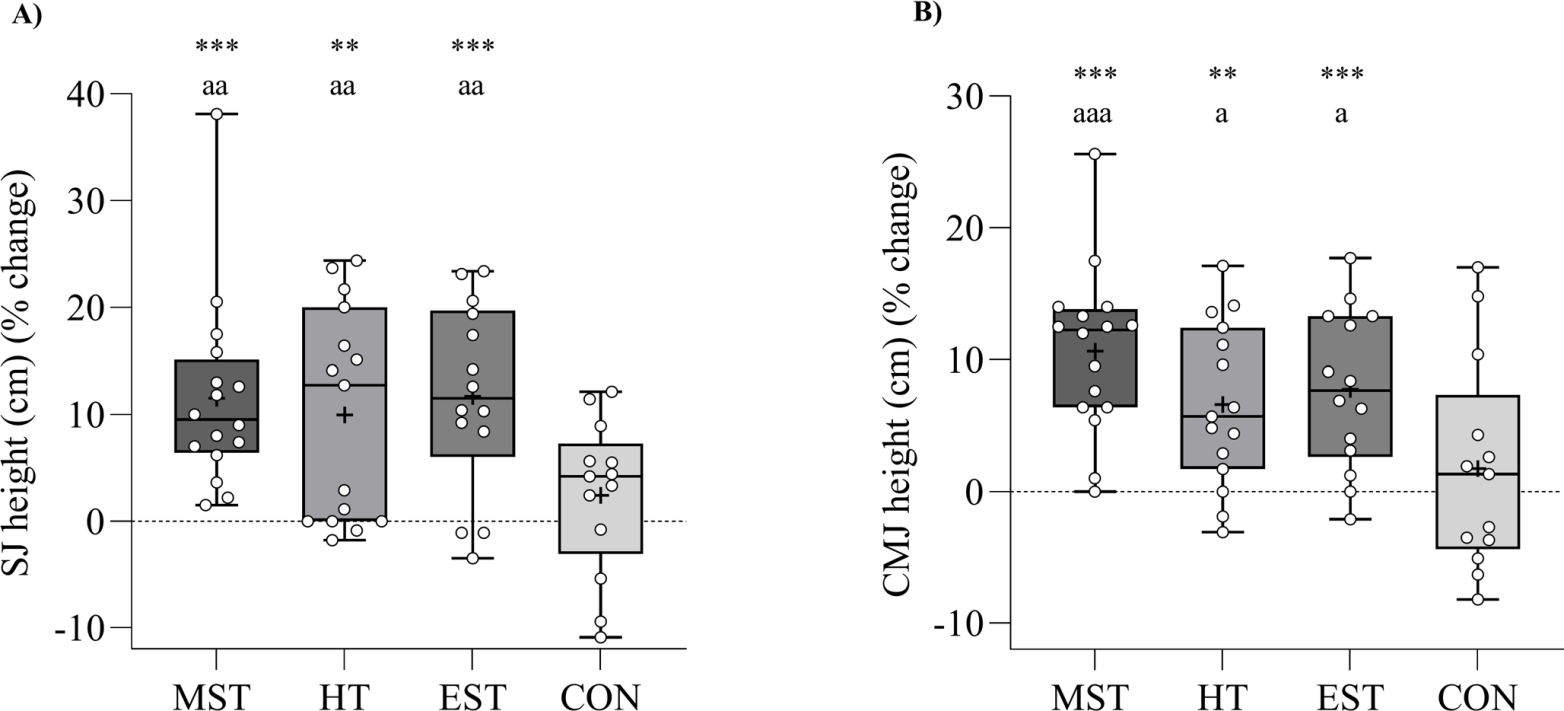
Percentage (%) change (∆) in squat jump (SJ) height (cm) (*left*) and counter movement jump (CMJ) height (cm) (*Right*) from pre- to post-test in all training groups. Box plots display individual values (○), mean (+), median (─), 1 st and 3rd quartiles, interquartile range, and minimum and maximum values. SJ, Squat jump, CMJ, Counter movement jump, MST, Maximal strength training; HT, Hypertrophy training; EST, Explosive strength training; CON, Control. Significant within-group ∆ from pre- to post-test (^***^p ≤ 0.001, ^**^p ≤ 0.01). Significant between-group difference in post-test value adjusted for pre-test value compared to CON (^aaa^ *p* ≤ 0.01, ^aa^ *p* ≤ 0.01, ^a^ *p* ≤ 0.05)

SJ-height (cm) improved more after MST, HT and EST compared to CON from pre- to post-test (all *p* ≤ 0.01, respectively). No differences in post-test SJ-height (cm), adjusted for pre-test values, were observed between MST, HT and EST. η² for the between-group comparisons was 0.180. Within groups, MST improved SJ-height (cm) by 2.2 ± 1.2 cm (*p* ≤ 0.001, *d* = 1.86, 95% CI [1.0, 2.7]), HT improved by 2.0 ± 2.1 cm (*p* ≤ 0.01, *d* = 0.96, 95% CI [0.3, 1.6]) and EST improved by 2.2 ± 1.9 cm (*p* ≤ 0.001, *d* = 1.17, 95% CI [0.5, 1.8]). No change was found in CON (Table 4; Fig. 4).

**Table 4:** Squat jump (SJ)- and counter movement jump (CMJ)-height at pre- and post-test

	MST (*n* = 16) 8 males and 8 females		HT (*n* = 15) 9 males and 6 females		EST (*n* = 14) 7 males and 7 females		CON (*n* = 13) 5 males and 8 females	
	Pre-test	Post-test	Pre-test	Post-test	Pre-test	Post-test	Pre-test	Post-test
SJ-height (cm)	20.9 ± 5.0	23.0 ± 4.9^***^^aa^	22.4 ± 5.3	24.4 ± 5.4^**^^aa^	19.9 ± 5.1	22.0 ± 5.7^***^^aa^	20.2 ± 5.6	20.6 ± 5.0
CMJ-height (cm)	23.1 ± 6.3	25.4 ± 6.4^***^^aaa^	25.0 ± 5.6	26.6 ± 6.3^**^^a^	23.3 ± 5.4	25.1 ± 5.6^***^^a^	22.9 ± 6.5	23.1 ± 6.1

Data are presented as mean ± SD. SJ: Squat jump; CMJ: Counter movement jump; MST: Maximal strength training; HT: Hypertrophy training; EST: Explosive strength training; CON: Control

Significant within-group ∆ from pre- to post-test (^***^p ≤ 0.001, ^**^p ≤ 0.01). Significant between-group difference in post-test value adjusted for pre-test value compared to CON (^aaa^ *p* ≤ 0.001, ^aa^ *p* ≤ 0.01, ^a^ *p* ≤ 0.05)

CMJ-height (cm) improved more after MST, HT and EST compared to CON from pre- to post-test (*p* ≤ 0.001, *p* ≤ 0.05 and *p* ≤ 0.05, respectively). No differences in post-test values, adjusted for pre-test values, were observed between MST, HT and EST. η² for the between-group comparisons was 0.210. Within groups, MST improved CMJ-height (cm) by 2.3 ± 1.3 cm (*p* ≤ 0.001, *d* = 1.81, 95% CI [1.0, 2.6]), HT improved by 1.6 ± 1.7 cm (*p* ≤ 0.01, *d* = 0.94, 95% CI [0.3, 1.5]) and EST improved by 1.7 ± 1.3 cm (*p* ≤ 0.001, *d* = 1.30, 95% CI [0.6, 2.0]). No change was found after CON (Table 4; Fig. 4).

### Correlations

Pre-test squat 1RM was associated with pre-test RFD_SJ50%_ (*r* = 0.61), RFD_SJ30%_, (*r* = 0.64), RFD_SJ0%_ (*r* = 0.77), PF_SJ50%_ (*r* = 0.87), PF_SJ30%_ (*r* = 0.83) and PF_SJ0%_ (*r* = 0.76), respectively (all *p* ≤ 0.001). Pre-test squat 1RM was also associated with pre-test SJ-height (*r* = 0.53) and CMJ-height (*r* = 0.56) (all *p* ≤ 0.001). Associations were found between ∆squat 1RM and ∆RFD_SJ50%_ (*r* = 0.30, *p* ≤ 0.05), ∆RFD_SJ30%_ (*r* = 0.48, *p* ≤ 0.001), ∆RFD_SJ0%_ (*r* = 0.35, *p* ≤ 0.01), ∆PF_SJ30%_ (0.34, *p* ≤ 0.01) and ∆PF_SJ0%_ (*r* = 0.32, *p* ≤ 0.05), respectively, from pre- to post-test. Moreover, associations were found between ∆squat 1RM and ∆SJ-height (*r* = 0.41) and ∆CMJ-height (*r* = 0.55) (all *p* ≤ 0.001) from pre- to post-test. The absolute ∆squat 1RM in MST and HT pooled correlated with ∆SJ-height (*r* = 0.37, *p* ≤ 0.05) and ∆CMJ-height (*r* = 0.55, *p* ≤ 0.05), but this association was absent in EST (SJ: *r* = −0.045, *p* = 0.878; CMJ: *r* = 0.059, *p* = 0.840).

## Discussion

The main findings of the current study were that (1) Velocity-specific EST was effective to improve unloaded jump performance, leading to similar improvements in SJ- and CMJ-height as MST and HT. (2) MST were more effective than EST to improve lower-body explosive strength against moderate loads (RFD_SJ50%−SJ30%_). (3) MST and HT were more effective than EST to improve maximal strength. While the first main finding is considered exploratory, the second and third represent confirmatory findings based on our predefined primary outcomes and corresponding hypotheses. Together, lower-body EST seems to provide an effective task-specific stimulus to improve SJ- and CMJ-height, whereas strength training with high loads (≥ 70% of 1RM) leads to improvements in explosive performance across a wider loading-spectre. Compared to the upper-body data of Trane et al. (2025) the current findings suggest a more pronounced load- and velocity-specific training effect in squat jumps than in bench press.

### Lower-body maximal strength - Squat 1RM

A key confirmatory outcome was that squat 1RM improved more after MST (+ 20.7%) and HT (+ 18.2%) compared to EST (+ 10.9%). This pattern is consistent with our recent upper-body study within the same sample, in which MST (+ 21.5%) and HT (+ 17.9%) improved bench press 1RM more than EST (+ 5.8%) (Trane et al. 2025). These data add to considerable evidence suggesting that strength training with high loads (≥ 70% of 1RM) is more effective than training with low loads (0–50% of 1RM) to improve maximal strength (Campos et al. 2002; Cormie et al. 2007a, 2010a; Harris et al. 2000, 2008; Jones et al. 2001; McBride et al. 2002; Wilson et al. 1993).

MST has often been demonstrated to improve maximal strength more than HT (Campos et al. 2002; Heggelund et al. 2013; Schoenfeld et al. 2016). In this study, MST did not yield larger improvements in 1RM than HT, although MST exhibited larger effect size (*d* = 3.40 vs. 1.51). Moreover, the improvements in 1RM after MST in the current study is within the lower range of previous reports (+ 18%−61%) (Campos et al. 2002; Heggelund et al. 2013; Kittilsen et al. 2021; Schoenfeld et al. 2016; Tøien et al. 2018), which may also explain the lack of difference between MST and HT. Nevertheless, our data highlights that also HT can be highly effective to increase maximal strength. Considering training volume as a potent driver of maximal strength (Ratamess et al. 2009), the lack of difference in 1RM response between MST and HT may be a result of the 1.5–2.5 times greater workload in HT. In line with this notion, Heggelund et al. (2013) volume-matched MST and HT and reported greater percentage improvements in 1RM following MST (+ 50%) compared to HT (+ 35%). However, differences in workload between HT and MST/EST make training volume a major unresolved confounding factor when interpreting between-group differences in maximal strength adaptations in the present study.

### Explosive strength - Rate of force development in squat jump at 50%, 30% and 0% of 1RM

The magnitude of improvements in RFD_SJ50%−SJ0%_ after MST (+ 12.3–21.7%) and HT (+ 11.4–14.3%) coincide with the upper-body study (Trane et al. 2025) and suggest that RFD is effectively improved by MST and HT, in line with previous literature (Aagaard et al. 2002; Cormie et al. 2010a; Haglo et al. 2022; Heggelund et al. 2013; Helgerud et al. 2020; Støren et al. 2008; Tøien et al. 2018; Unhjem et al. 2015). Contrary to our hypothesis, MST did not yield larger RFD-improvements than HT, although effect sizes slightly favoured MST (*d* = RFD_SJ50%_: 1.27 vs. 1.06; RFD_SJ30%_: 1.38 vs. 0.98; RFD_SJ0%_:1.08 vs. 0.62, for MST and HT respectively).

Conflicting evidence exists on whether high loads (≥ 70% of 1RM) or low loads (0–50% of 1RM) are most effective to enhance RFD in the loading range between 0 and 50% of 1RM (Cormie et al. 2010a; Newton et al. 1999). Our data suggest that high loads are more effective, particularly when aiming to enhance performance against moderate loads, as MST increased RFD_SJ50%_ (+ 22% vs. 11%) and RFD_SJ30%_ (+ 18% vs. 7%) more than EST. Also, HT was more effective than EST to improve RFD_SJ30%_ (+ 14% vs. 7%). In contrast, the results for RFD_SJ0%_ were less clear. As depicted in Fig. 3C, the improvements after EST were like the improvements after MST and HT (all within + 10–12%), although large within-group variability led to non-significant results for EST. Moreover, since none of the strength training groups increased RFD_SJ0%_ significantly more than CON, definite conclusions cannot be made. The absence of any significant between-group differences, and no within-group improvements found in RFD_SJ0%_ following EST may be attributed to methodological factors. Specifically, we observed greater variability in the RFD_SJ0%_ assessments compared to RFD_SJ50%_ and RFD_SJ30%_ (CV = 9% vs. 6%), as well as SJ height assessments using the impulse-momentum method, which has been consistently reported to display low variability (CV = < 5%) (Dal Pupo et al. 2025; Eythorsdottir et al. 2024). Collectively, the greater variability in our unloaded RFD_SJ0%_ assessments, compared to the loaded RFD_SJ50−SJ30%_ condition and SJ height assessments, may have influenced the observed outcomes.

### Squat jump (SJ)- and counter movement jump (CMJ)-height

Although EST was not effective to increase RFD_SJ50%−SJ0%,_ it did increase SJ- and CMJ-height to a similar extent as MST and HT. This load- and velocity-specific adaptation contrasts the upper-body data of Trane et al. (2025), in which EST performed at 40% of 1RM was less effective than MST to improve bench press mean propulsive velocity at 40% of 1RM. The discrepancy between squat and bench press responses may be taken as evidence that velocity-specificity in strength training is more pronounced in the lower vs. upper body. We speculate that this may be related to differences in the relative importance of coordination and movement technique between the two exercises. The complexity of explosive SJs, which is a whole-body movement involving triple extension of ankle, knee, and hip allows for controlling more degrees of freedom (Cormie et al. 2011; Dal Pupo et al. 2025), and requires stable control of the centre of mass within a closed kinetic chain (Prokopy et al. 2008; Dal Pupo et al. 2025). Consequently, this may lead to distinct muscle activation patterns (Behm et al. 2025; Behm & Sale., 1993b) and require more advanced coordinative skills and athletic abilities compared to the bench press movement, which is performed in an open kinetic chain (Prokopy et al. 2008; Suchomel et al. 2018). Thus, we speculate that practising jumping may have a larger effect on jump height relative to MST, than what could be expected from explosive bench press throws at 40% of 1RM vs. MST. As expected, we observed correlations within the two heavy strength training groups (MST and HT pooled) between ∆squat 1RM and ∆jump height (∆SJ: *r* = 0.37, *p* ≤ 0.05; ∆CMJ: *r* = 0.55, *p* ≤ 0.05), indicating that gains in maximal strength transfers effectively into explosive athletic performance. Interestingly, this correlation was completely absent within EST (∆SJ: *r* = −0.045, *p* = 0.878; ∆CMJ: *r* = 0.059, *p* = 0.840), in turn indicating that other factors may be influential to the increase in jump height following EST (Cormie et al. 2010a). As elegantly phrased by Almåsbakk and Hoff (1996) coordination may represent an important determinant of velocity-specific adaptations to novel unfamiliar tasks, suggesting that improved explosive performance may not only relate to ‘velocity-specific force generating capacity per se’. The same topic was recently reviewed by Behm et al. (2025), who suggested that practicing the specific coordination pattern and ‘skill’ you want to develop represent an effective strategy to improve explosive performance, especially in novel and complex lower-body tasks with high coordination requirements. We agree with this perspective and find it likely that the more pronounced velocity-specificity in squats compared to bench press reflects different levels of motor control demands between the two exercises. However, this notion remains speculative, as only SJ- and CMJ-height, as well as RFD_SJ50%−SJ0%_ were assessed, and no coordination measurements (e.g., EMG or kinematic recordings) were conducted in the present study to further support this claim.

### Lower-body muscular hypertrophy and body weight

Exploratory findings showed greater improvements in leg lean mass in HT compared to EST. Moreover, both MST and HT increased leg lean mass by 3.1% and 3.7%, respectively, while EST did not change leg lean mass from pre- to post-test. These findings suggest that lower-body muscular hypertrophy can be effectively achieved through strength training between 70 and 95% of 1RM, in line with previous literature (Campos et al. 2002; Schoenfeld et al. 2016, 2017a, b, 2021a, b; Vissing et al. 2008). In contrast, lower-body EST seems to have little effect on muscular hypertrophy. The substantially greater intensity (% of 1RM) and duration of concentric loading (ms) in squat MST and HT (≥ 70% of 1RM and > 500 ms) compared with unloaded SJ EST (0% of 1RM and < 300 ms) represents some potential explanations to why MST and HT induced lower-body hypertrophic adaptations while EST did not (Balshaw et al. 2016; Fry 2004; Schoenfeld. 2020, pp.30–31). Notably, the ~ 0.5 kg increase in leg lean mass after MST may indicate that the high mechanical tension stimulus during MST represents a potent stimulus for muscular hypertrophy, despite being performed at a lower total training volume compared to HT (Mangine et al. 2015; Ratamess et al. 2009; Schoenfeld 2010; Schoenfeld et al. 2021a). However, the importance of training volume as a potent driver of muscular hypertrophy complicates direct comparisons between methods (Schoenfeld 2010; Schoenfeld et al. 2017a, b), particularly since HT was performed at ~twofold higher workloads compared to MST and EST, a factor that could bias hypertrophy findings in favour of HT compared to MST and EST.

Athletes participating in sports that demands high levels of relative strength, often perceive increases in body weight to be undesirable. HT increased body weight by 1.1 kg, while body weight remained unchanged after MST and EST. Newton’s second law of motion states that acceleration is the product of force divided by the mass the force acts upon. Hence, increased muscular hypertrophy and body weight after HT could theoretically cause deteriorated acceleration in explosive tasks, if force generating capacity is unchanged. However, the improvement in SJ- and CMJ-height, coupled with large improvements in 1RM and RFD_SJ50%− SJ0%_ after HT, by far outweighed the negative impact of increased body weight. Thus, if force generating capacity is effectively improved after HT, there is no need to be concerned about the negative consequences of muscular hypertrophy and increased body weigh in the short term. However, we cannot ignore that longer-term HT (i.e., more than 8 weeks) may lead to undesirable increases in body weight.

### Practical considerations

The findings of the present study offer novel insight into how professionals working with sports, rehabilitation, or general physical fitness and health can manipulate the interdependent programming variables of external load and concentric velocity to target specific strength training adaptations. The results suggest that high-load MST or HT trained between 70 and 95% of 1RM, remains essential for enhancing lower-body maximal and explosive strength, while also improving jump performance to a similar extent as low-load EST. This highlights the importance of not neglecting high-load strength when aiming for improved performance in low-load and high-velocity tasks.

In contrast to the upper-body data of Trane et al. (2025), we observed significant load- and velocity-specific training adaptations in response to lower-body EST, which seemed to occur quite independent of changes in force generating capacity. Although somewhat speculative, this marked discrepancy between squat and bench press responses may suggest that the more coordinatively demanding the exercise, the more benefit can be derived from practising the task you want to improve. Importantly, our results also demonstrate that lower-body explosive performance can be effectively enhanced by MST and HT, and that this is likely due to improved force generating capacity.

Behm et al. (2025) forwarded the notion that strength training with high loads and maximal intended velocity (e.g., MST), in combination with specific low load - high velocity training (e.g., EST) may represent an especially effective approach to improve explosive athletic performance. In real world athletic programming, this combination is quite common across different sports (Cormie et al. 2011; Haff and Nimphius 2012; Suchomel et al. 2018). Although we have not examined such an approach in the current studies, it appears likely that the combination of MST and EST may offer complementary adaptations, leading to improvements in both force generating capacity as well as task specific motor control. For future studies, it would be interesting to examine whether a combination of MST and EST, referred to as a mixed methods approach (Haff and Nimphius 2012), may improve explosive performance more than MST or EST alone. Based on the findings of others (Cormie et al. 2007a; Harris et al. 2000), we suggest that practitioners aiming to enhance explosive lower-body athletic performance, such as athletes in team sports (e.g., football, European handball), among others, could benefit from adopting a mixed method strength training approach along with their sport-specific training. Specifically, by combining high-load MST or HT with low-load EST as a key supplemental training stimulus, together with training for their specific sport practice. This approach effectively targets various segments of the load-velocity profile, and we speculate that it may result in more favourable overall adaptations by improving coordinative abilities at high velocities, as well as force application against both high and low loads.

It is important to consider the subject’s strength training status (Squat 1RM/Body weight ratio: 1.32) when interpreting our data, since strength level may influence the response to different training methods (Cormie et al. 2010b; Suchomel et al. 2016; Wetmore et al. 2020). While our data propose that strength gains following MST and HT are effectively transferred into improved athletic performance at high velocities, these findings are primarily valid for non-strength trained individuals. According to Suchomel et al. (2016), the potential to improve athletic performance by increasing force generating capacity may be diminished at high strength levels (Squat 1RM/Body weight ratio: >2.0). Approaching this strength level, further increases in strength becomes both more difficult to achieve and may also have less transfer to explosive performance (Suchomel et al. 2016). At this point, practicing the exercise/movement in which you aim to optimize performance, may become increasingly rewarding compared to heavy strength training. Thus, strength trained subjects may possibly benefit relatively more from EST vs. MST than the non-strength trained subjects in the present study (Squat 1RM/Body weight ratio: 1.32).

Notably, data from this heterogenous mixed-sex sample demonstrated substantial inter-individual variability in strength training adaptations to MST, HT and EST, especially in RFD_SJ50%−SJ0%_, SJ- and CMJ-performance. While some subjects demonstrated considerable improvements (often termed “responders”), others showed no change or even negative adaptations (often termed “non-responders”). Hence, we cannot rule out the possibility that differences in inter-individual responses to different types of strength training may have affected the overall group-level effects in the present study. This variability may be attributed to individual baseline characteristics, such as initial strength training status, jumping skill level, sex, among other factors (Cormie et al. 2010b; Roberts et al. 2020; Suchomel et al. 2016, 2018; Wetmore et al. 2020). For example, the inclusion of both sexes may have introduced sampling bias if sex-based differences in previous strength training experience resulted in lower baseline strength for one sex relative to sex-specific normative values, potentially influencing adaptations in both maximal and explosive strength characteristics (e.g., RFD_SJ50%−SJ0%_, SJ- and CMJ-performance) (Kittilsen et al. 2021; Kojić et al. 2021; Miller et al. 1993; Nuzzo 2023; Roberts et al. 2020).

### Methodological considerations

In the present exploratory interventional trial, multiple hypotheses were tested both within and between groups. To ensure sufficient statistical power and reduce the risk of Type II errors (i.e., false negatives) for between-group comparisons, ANCOVAs were followed by Fisher’s LSD post hoc tests instead of more conservative alternatives (e.g., Bonferroni correction). However, this statistical approach may increase the risk of Type I errors (i.e., false positives), especially when applied to exploratory outcome variables outside the present study’s confirmatory outcomes and predefined hypotheses. Thus, because Fisher’s LSD was applied to all outcome variables, the risk of false positives should be considered when interpreting the findings. Also, it should also be noted that the per-protocol analysis conducted in the present study, after the dropout or exclusion of sixteen participants due to injury or other reasons, may have introduced selection bias, which could affect the generalizability of our findings. Moreover, care should be taken when interpreting our findings in relation to the expected long-term effects of the respective strength training methods examined in this study. For example, longer-term strength training protocols of more than eight weeks may elicit even more meaningful alterations in body weight and/or muscular hypertrophy (Schoenfeld, 2021b). In contrast, variables that rely more heavily on short-term neural adaptations (e.g., jumping height) may plateau more rapidly following strength training, as the point of diminishing returns is reached (Cormie et al. 2011; Kraemer and Newton 2000). In addition, because the study was initially designed to mirror real-world applications of the respective strength training methods, MST and EST were consequently performed with lower workloads (i.e., training volume) compared to HT, which should be considered when interpreting between-group adaptations.

All groups in the present study, including CON, completed two weekly sprint training sessions as part of the program. These sprints were introduced to investigate the effects of combining sprints with different strength training methods on sprint performance and other complex athletic performance metrics, which will be presented in an upcoming paper. As these sprint efforts involved high-velocity muscle actions and a high RFD, we cannot eliminate the possibility that the inclusion of short sprints may have reduced the dissimilarities between high-load strength training (MST and HT) and low-load EST, compared to if these methods had been trained in isolation without sprint training.

Finally, since our sample included both male and female subjects, we cannot exclude the possibility that sex-based differences in training responses may have influenced our results. For example, as shown in Table 1, males typically display numerically greater pre-training lower-body maximal strength than females (Kittilsen et al. 2021; Kojić et al. 2021; Miller et al. 1993; Nuzzo 2023; Roberts et al. 2020). These differences are commonly attributed to factors such as larger body size, greater muscle mass, and larger type II muscle fibre areas in males, as well as hormonal differences between sexes, all of which may influence strength training responses (Kittilsen et al. 2021; Miller et al. 1993; Nuzzo 2023; Roberts et al. 2020). Males typically demonstrate numerically greater absolute improvements in lower-body maximal strength compared to females, whereas relative improvements (∆%) have often been reported to be similar between sexes, although this is not always a consistent finding (Kittilsen et al. 2021; Kojić et al. 2021; Nuzzo 2023; Roberts et al. 2020). Adaptations of explosive performance characteristics (e.g., RFD_SJ50%−SJ0%_ or SJ- and CMJ-height) and muscular hypertrophy may also follow similar sex-specific patterns as maximal strength improvements, given their many shared determinants (Maffiuletti et al. 2016; Nuzzo 2023; Schoenfeld et al. 2021b; Suchomel et al. 2016, 2018).

Nevertheless, the sample size in the present study was insufficient to address potential sex-based differences in training responses, and the study did not control for oral contraceptive use or menstrual cycle phase in female participants. Hence, our findings do not clarify whether strength training prescriptions should differ between males and females and should be interpreted with caution to avoid implicit generalization across sexes. However, equal allocation into training groups based on sex was performed to limit the influence of sex-based differences on group-level analyses, and we considered the potential drawbacks of including both sexes to be outweighed by the importance of increasing female representation in exercise physiology research (James et al. 2023). Moreover, and perhaps most importantly, both sexes appear to show great potential for strength training-induced adaptations (Kittilsen et al. 2021; Kojić et al. 2021; Nuzzo 2023; Roberts et al. 2020).

## Conclusions

In contrast to the identically designed upper body study by Trane et al. (2025), lower-body EST provided a potent load- and velocity-specific training stimulus, as evidenced by exploratory findings of similar improvements in SJ- and CMJ-performance as those observed after MST and HT. While improvements in jump height following MST and HT seem to rely on adaptive changes in force generating capacity, improved jump height following EST appears to originate from other mechanisms not directly measured in this study.

## Supplementary Information

Supplementary Material 1.

## Data Availability

The data sets generated during and/or analysed during the current study are available from the corresponding author on reasonable request.

## Abbreviations

ANCOVA: Analysis of covariance
ANOVA: Analysis of variance
1RM: One repetition maximum
CI: Confidence interval
CV: Coefficient of variation
CON: Control
CMJ: Counter movement jump
DXA: Dual x-ray absorptiometry
EST: Explosive strength training
HT: Hypertrophy training
ICC: Intraclass correlation coefficient
J: Joules
LSD: Least significant difference
MST: Maximal strength training
N: Newtons
N ∙ s^− 1^: Newtons per second
PF_SJ50%_: Squat jump peak force at 50% of squat 1RM
PF_SJ30%_: Squat jump peak force at 30% of squat 1RM
PF_SJ0%_: Squat jump peak force at 0% of squat 1RM
PPO: Peak power output
RFD: Rate of force development
RFD_SJ50%_: Squat jump rate of force development at 50% of squat 1RM
RFD_SJ30%_: Squat jump rate of force development at 30% of squat 1RM
RFD_SJ0%_: Squat jump rate of force development at 0% of squat 1RM
RIR: Repetitions in reserve
SJ: Squat jump
η²: Partial eta squared
