# Supplementary material for: Load- and velocity-specific adaptations to lower-body maximal strength training (MST), hypertrophy training (HT) and explosive strength training (EST)
Source: Eur J Appl Physiol. 2026 Feb 12;126(6):3323–41. doi: 10.1007/s00421-026-06156-2 (PMC13287104; doi:10.1007/s00421-026-06156-2)
Supplement: Supplementary file 1 — Supplementary Material 1. [file 421_2026_6156_MOESM1_ESM.docx]

Supplementary materials


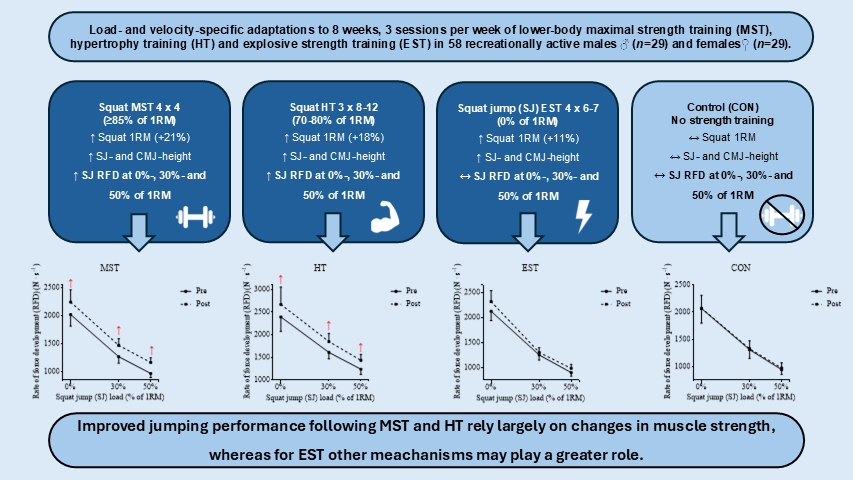


Supplementary figure 1. Graphical abstract of the study titled “Load- and velocity-specific adaptations to lower-body maximal strength training (MST), hypertrophy training (HT) and explosive strength training (EST)”. ↑ denotes a significant within-group change from pre- to post-test. Data are presented as mean ± SE.


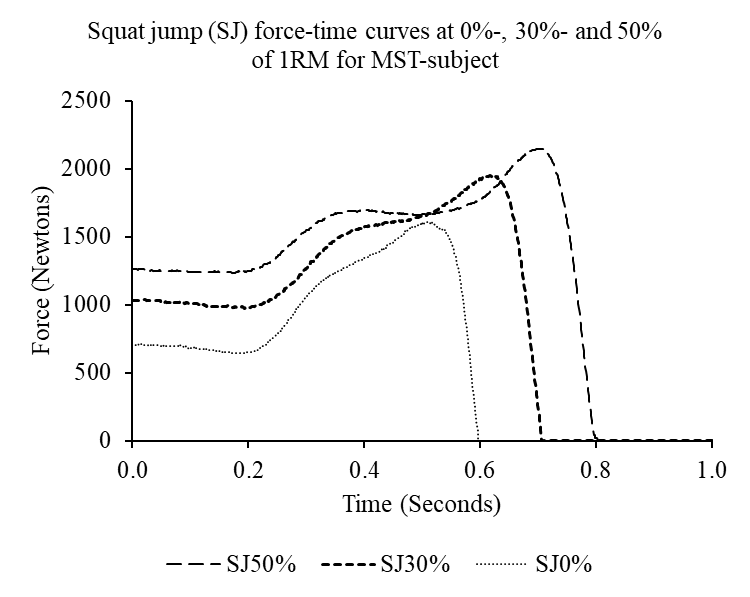


Supplementary figure 2. Squat jump (SJ) force-time curves at 0%-, 30%-, and 50% of 1RM for a representative male MST-subject.


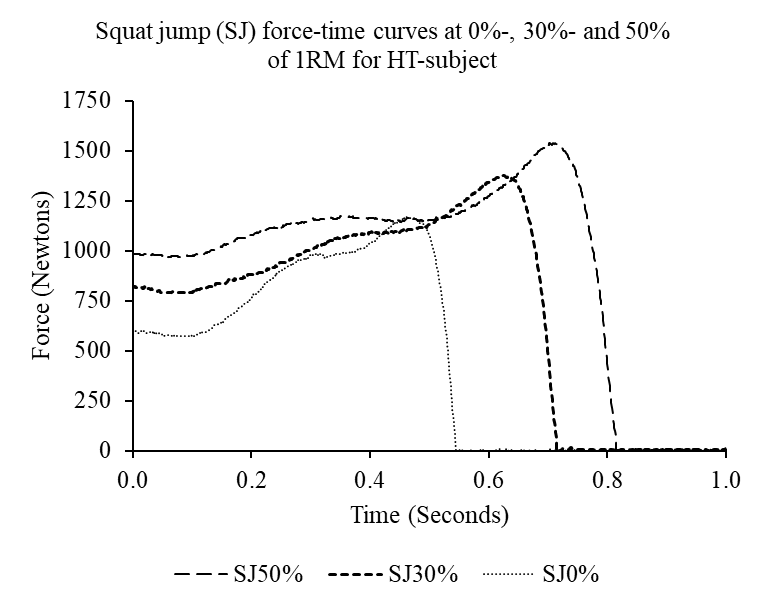


Supplementary figure 3. Squat jump (SJ) force-time curves at 0%-, 30%-, and 50% of 1RM for a representative female HT-subject.


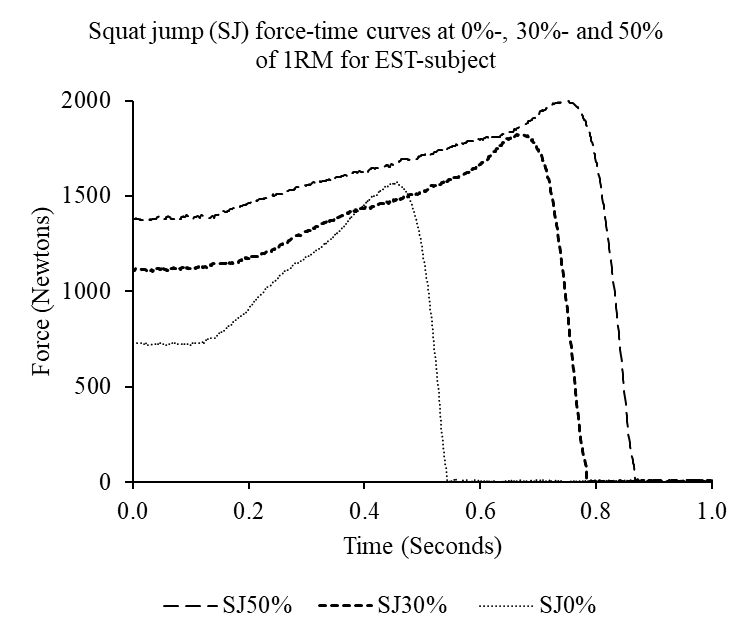


Supplementary figure 4. Squat jump (SJ) force-time curves at 0%-, 30%-, and 50% of 1RM for a representative male HT-subject.


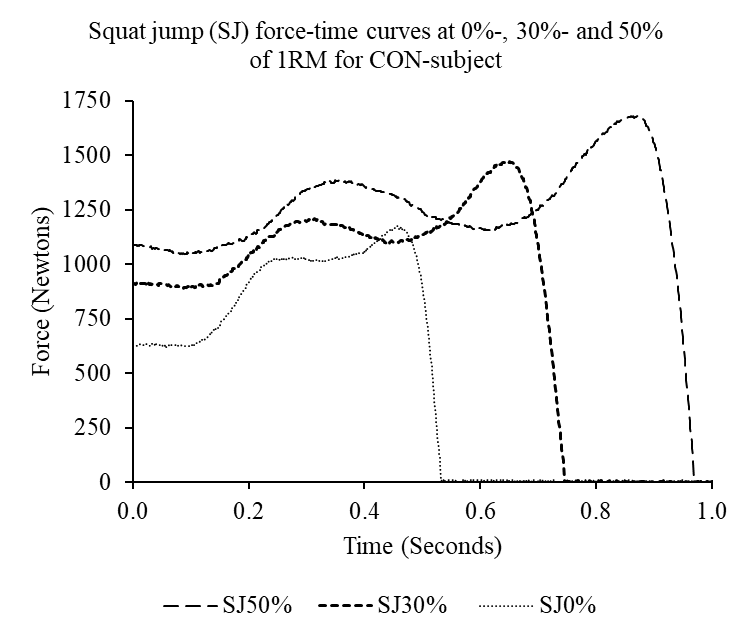


Supplementary figure 5. Squat jump (SJ) force-time curves at 0%-, 30%-, and 50% of 1RM for a representative female CON-subject.
